# Artificial Intelligence to Meet the Psychosocial Needs of Cancer Patients: A Systematic Review of Randomized Controlled Trials

**DOI:** 10.1192/j.eurpsy.2026.10222

**Published:** 2026-07-31

**Authors:** N. D. Kılınç, F. Oflaz

**Affiliations:** 1 Institute of Health Sciences; 2 School of Nursing, Koç University, İstanbul, Türkiye

## Abstract

**Introduction:**

Cancer diagnosis frequently leads to psychosocial distress, including anxiety, depression, and reduced quality of life. Although psychosocial support is essential, access barriers limit timely care. Artificial intelligence (AI) has emerged as a scalable and accessible means of delivering psychosocial interventions.

**Objectives:**

This study systematically reviewed randomized controlled trials (RCTs) evaluating AI-based interventions addressing psychosocial needs in cancer patients, focusing on intervention types, effectiveness, and implications for supportive care.

**Methods:**

Following PRISMA guidelines, a systematic review was conducted (PROSPERO: CRD420251134621). Database searches identified 996 records; 143 full texts were assessed, and 7 RCTs met inclusion criteria, encompassing 913 cancer patients. Eligible studies tested AI-based interventions designed to address unmet psychosocial needs. Risk of bias was assessed using the Cochrane RoB 2 tool.

**Results:**

Interventions included chatbots, mobile apps, digital counseling, and app-based therapeutics. Most demonstrated positive psychosocial effects, with significant reductions in anxiety, depression, and distress, particularly in younger patients and those receiving chatbot or app support. Additional benefits included improved self-efficacy, social support, knowledge, and empowerment. Some studies also reported reductions in fatigue and improved attitudes toward AI. While certain trials showed no significant effects on distress or quality of life, feasibility, acceptability, and patient satisfaction were consistently high. Secondary benefits included reduced pain, fewer pain-related hospitalizations, and better symptom management (Image 1).

**Image 1:**
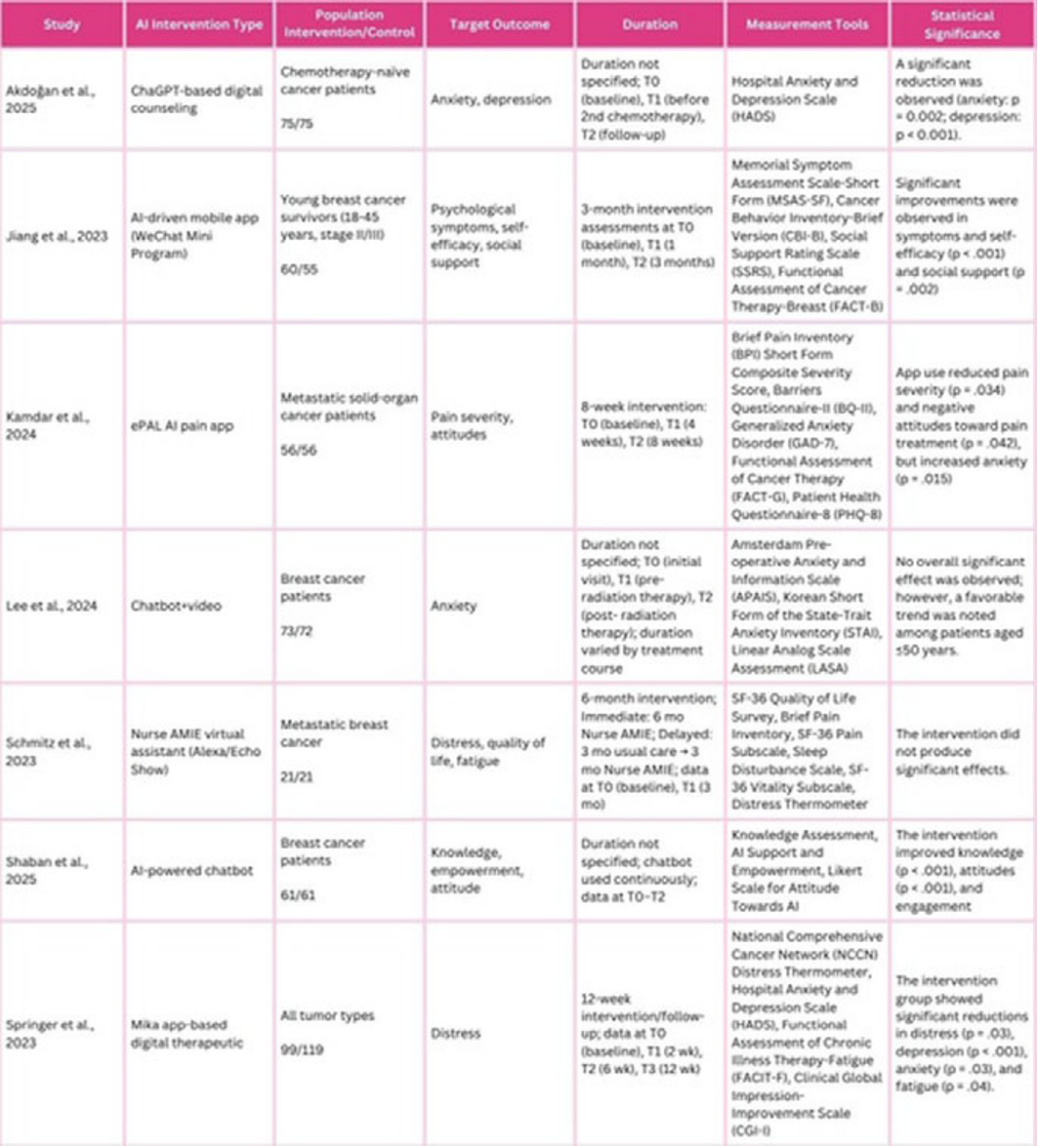
Image 1: Long description.

**Conclusions:**

RCT evidence indicates that AI-based interventions can effectively reduce psychosocial distress and related symptoms in cancer patients, while enhancing empowerment and self-management. Despite mixed effects on quality of life, consistent psychological and symptomatic benefits highlight their potential as valuable adjuncts to oncology care. Further large-scale, culturally diverse, and long-term trials are needed to confirm generalizability and sustainability.

**Disclosure of Interest:**

None Declared

## Long descriptions

### Image 1: Long description

A table with seven columns: Study, A I Intervention Type, Population Intervention/Control, Target Outcome, Duration, Measurement Tools, and Statistical Significance.

* Row 1: Akdoğan et al., 2025. ChatGPT-based digital counseling for 75/75 chemotherapy-naive cancer patients. Target: Anxiety, depression. Duration: T 0 to T 2. Tools: H A D S. Result: Significant reduction (anxiety p = 0.002; depression p < 0.001).

* Row 2: Jiang et al., 2023. A I-driven mobile app for 60/55 young breast cancer survivors. Target: Psychological symptoms, self-efficacy, social support. Duration: 3 months. Tools: M S A S-S F, C B I-B, S S R S, F A C T-B. Result: Significant improvements in symptoms and self-efficacy (p < .001) and social support (p = .002).

* Row 3: Kamdar et al., 2024. ePAL A I pain app for 56/56 metastatic solid-organ cancer patients. Target: Pain severity, attitudes. Duration: 8 weeks. Tools: B P I, B Q-I I, G A D-7, F A C T-G, P H Q-8. Result: Reduced pain severity (p = .034) and negative attitudes (p = .042), but increased anxiety (p = .015).

* Row 4: Lee et al., 2024. Chatbot+video for 73/72 breast cancer patients. Target: Anxiety. Duration: T 0 to T 2. Tools: A P A I S, S T A I, L A S A. Result: No overall significant effect; favorable trend for patients aged ≤ 50 years.

* Row 5: Schmitz et al., 2023. Nurse A M I E virtual assistant for 21/21 metastatic breast cancer patients. Target: Distress, quality of life, fatigue. Duration: 6 months. Tools: S F-36, B P I, Sleep Disturbance Scale, Distress Thermometer. Result: No significant effects.

* Row 6: Shaban et al., 2025. A I-powered chatbot for 61/61 breast cancer patients. Target: Knowledge, empowerment, attitude. Duration: T 0-T 2. Tools: Knowledge Assessment, Likert Scale. Result: Improved knowledge (p < .001), attitudes (p < .001), and engagement.

* Row 7: Springer et al., 2023. Mika app-based digital therapeutic for 99/119 patients with all tumor types. Target: Distress. Duration: 12 weeks. Tools: N C C N Distress Thermometer, H A D S, F A C I T-F, C G I-I. Result: Significant reductions in distress (p = .03), depression (p < .001), anxiety (p = .03), and fatigue (p = .04).

Navigate back to Image 1:.

